# Editorial: Proceedings of ABBS-international conference on biohydrogen and bioprocesses 2022 (ABBS 2022)

**DOI:** 10.3389/fbioe.2024.1390377

**Published:** 2024-04-09

**Authors:** Ao Xia, Christiane Herrmann, Alissara Reungsang, Pau-Loke Show, Eric Trably, Junjun Wu

**Affiliations:** ^1^ Key Laboratory of Low-grade Energy Utilization Technologies and Systems, Chongqing University, Ministry of Education, Chongqing, China; ^2^ Institute of Engineering Thermophysics, School of Energy and Power Engineering, Chongqing University, Chongqing, China; ^3^ Leibniz Institute for Agricultural Engineering and Bioeconomy (ATB), Potsdam, Germany; ^4^ Research Group for Development of Microbial Hydrogen Production Process from Biomass, Department of Biotechnology, Faculty of Technology, Khon Kaen University, Khon Kaen, Thailand; ^5^ Department of Chemical Engineering, Khalifa University, Abu Dhabi, United Arab Emirates; ^6^ Department of Chemical and Environment Engineering, Faculty of Science and Engineering, University of Nottingham Malaysia, Semenyih, Malaysia; ^7^ INRAE, Université de Montpellier, LBE, Narbonne, France

**Keywords:** biofuels, waste treatment, fermentation, mass transfer, circular economy

A massive amount of solid wastes (such as straws, manures, plastic and food wastes), liquid wastes (such as municipal and agricultural wastewaters), and gaseous wastes (such as flue gases rich in carbon dioxide) are generated from industrial, residential, transportation and agricultural sectors (Siegelman et al., 2021; Vyas et al., 2022; You et al., 2022). These wastes would cause significant adverse environmental impacts if they are not properly treated. Turning wastes into biofuels and bioproducts can make a substantial contribution to the clean energy supply and environmental protection (Bhatia et al., 2018; Peng et al., 2023; Wang et al., 2023). However, competitive and sustainable circular economy solutions via scientific and technological innovation are urgently needed to improve the conversion rates and efficiencies of such processes. The Research Topic includes four original research papers, which could provide various solutions to upgrade the solid, liquid, and gaseous wastes to value-added products.

Anaerobic digestion is a mature technology and has been developed for over a hundred years. However, anaerobic mono-digestion suffers from drawbacks such as low biogas/biomethane yield, digester instability and limited year-round availability of specific feedstocks (Karki et al., 2021). A study by Kriswantoro et al. investigated the performance of anaerobic co-digestion of untreated Napier grass and food waste hydrolyzed with subcritical water. An optimal biomethane yield of 614.37 mL/g VS was obtained at a 1:1 ratio (VS basis) of Napier grass and food waste during two-stage anaerobic digestion that sequentially produced biohydrogen and biomethane. The methane concentration in the biogas was higher than 65% on day 20 and maintained in the range of 65%–80% until the end of anaerobic digestion. Liquid digestate derived from anaerobic digestion contains high levels of nitrogen and phosphorus pollutants, which may require an energy-intensive and high-cost post-treatment process. The cultivation of microalgae may provide an alternative solution to treat liquid digestate while producing high-value biomass (Xia and Murphy, 2016). The work of Wang et al. assessed the potential of cultivation of microalgae *Chlorella* sp. in the liquid digestate from anaerobic digestion of brewer’s grains and brewery wastewater. A maximum biomass concentration of 1.36 g/L was obtained at 10% of digestate and 20% of brewery wastewater, which was 24.77% higher than the control cultivated in the BG11 medium. The maximum removal of ammonia nitrogen, chemical oxygen demand, total nitrogen, and total phosphorus achieved 98.20%, 89.98%, 86.98%, and 71.86%, respectively, suggesting a potential approach for liquid digestate treatment and microalgae biomass production.

Plastic waste has become a serious environmental issue in recent years; it is forecasted that plastic waste will rapidly accumulate to approximately 12 billion metric tons by 2050 (Geyer et al., 2017). The most promising route to tackle such a global issue is to turn plastic waste into small-molecule chemicals, which is considered as chemical upcycling. Lin et al. explored the feasibility of the synthesis of vitrimers (a class of covalent adaptable network plastic materials) with self-repairing properties by using plastic wastes as feedstocks. The raw materials were subjected to glycolysis to obtain the glycolysis products that were subsequently used as a reagent for the vitrimer synthesis process. The glycolysis of raw material and vitrimer synthesis processes were optimized.

Microalgae photosynthesis may offer an alternative solution to capture CO_2_ in ambient (420 ppm) or flue gas levels (10%–25%) (Chen and Xu, 2021), while it is important to improve the CO_2_ mass transfer at the gas-liquid interface. Zhao et al. proposed a new aeration device with bubble-cutting slices, which can separate bubbles into smaller sizes after their departure and improve the CO_2_ mass transfer. When the photobioreactor was equipped with the bubble-cutting slices, the bubble size and rising velocity decreased by 27.97% and 46.88%, respectively, while the prolongation of bubble residence time increased by 84.55%. Consequently, the dry weight and biomass productivity of microalgae *Chlorella pyrenoidosa* were improved by 6.99% and 33.33%, respectively. The authors demonstrated an interesting way to improve the CO_2_ mass transfer and enhance microalgae cultivation.

In conclusion, these works show various potential approaches to efficiently convert solid, liquid, and gaseous wastes to biofuels and bioproducts to achieve a circular economy. We would like to sincerely acknowledge all authors and reviewers for their important contributions to this Research Topic. We also would like to thank the Frontiers in Bioengineering and Biotechnology editorial team for their continued support and assistance.
